# Supplementary material for: “I would watch her with awe as she swallowed the first handful”: A qualitative study of pediatric multidrug-resistant tuberculosis experiences in Durban, South Africa
Source: PLoS One. 2022 Sep 16;17(9):e0274741. doi: 10.1371/journal.pone.0274741 (PMC9481007; doi:10.1371/journal.pone.0274741)
Supplement: S1 File — (DOCX) [file pone.0274741.s001.docx]

Annex 1: Interview Guides

Annex 1:

**INTERVIEW/FOCUS GROUP DISCUSSION GUIDE FOR CHILDREN**

**Group into 6-10 years and 11-14 years**

**FACILITATOR NAME & SURNAME: ____________________________________**

**CO-FACILITATOR NAME & SURNAME: ____________________________________**

**SCRIBE NAME & SURNAME: ____________________________________________**

**Number of children in the group: ___________**

**Age group of children: ___________**

**Ensure that a CONSENT FORM and REGISTER is filled in and signed by the caregivers, for each child, BEFORE the children are interviewed.**

Good day children. Welcome to our focus group meeting to improve the management of children with tuberculosis. My name is ***(Facilitator Name)***. I will be facilitating this session. Let me introduce you to ***(name of co-facilitator)*** who will be assisting me. This is ***(name of scribe)*** who will be writing some of the info down.

Is everyone comfortable with us speaking in isiZulu?

YES: ________ NO: ________ *If all yes, then speak in isiZulu. If some no, then speak in both isiZulu and English.*

Please feel comfortable. There are no wrong answers and we want you to speak openly and freely. Whatever you tell us here today is private, and only The Health Ninja team will know.

**Do you know what a Health Ninja is?** A Health Ninja is a **superhero** who fights for the health of themselves and their friends and families around them, by sharing important and correct information to everyone.

We will not share your stories with anyone else, like the doctors or your parents.

We need you to give us your honest opinions and experiences. Your stories and answers will help The Health Ninja and other children to fight of the BIG BAD TB Germs that you have managed to fight! How are you all feeling?

Please note that we will be taping the discussions for our team of Superhero’s to listen to, so we can help more little heroes like you. Remember the Coronavirus is still around, so please make sure that you always use your mask, covering your nose and mouth and remain at least 1.5m apart.

1. **First, let us introduce ourselves.** You don’t have to give us your real name. You can tell us a cool name that you want to be called today, so you can also be a celebrity for today.

*My name is* ***Ninja_________________,*** *what is your name?*

1. **Who knows why they are here today and what we are going to talk about?**
2. **Do you know why you had to come into hospital and take medicines?**
   1. Did your caregiver tell you what you were sick with?
   2. What do you know about the TB germ?
   3. How did you feel about having this DR-TB germ inside of you?
3. **Tell me about the medicines that you had to take or still have to take every day?**
   1. Show them the medicine and ask them to tell you how and when they took the medications?
   2. Who helped you to take the medicine?
   3. How did they help you?
4. **What is the hardest part about taking these medications?**
   1. Is there anything you don’t like about taking these medications?
   2. Why is this (If the group does not provide an answer then ask them about the taste, colour, smell or having to eat first, etc)?

1. **Is there anything you like about taking these medications?**
   1. Taste, colour, nice after taste, etc?
   2. Sitting on mom’s lap to take the medicine?
   3. Being allowed extra privileges if you took the medicines (can watch TV, go play, phone, etc)
2. **What do you think would make the medications better and easier to take?**
   1. Better Taste
   2. Take medicine at night
   3. Take medicine on an empty or full stomach
   4. Take it with yogurt or something to make the taste better
   5. If the tablets were smaller?
3. **Would you rather swallow tablets or have drink a liquid as medication?**

**USE THE MEDICINE BOARDS PROVIDED**

| **Medicine** | **Most common ANS** | **Why/Reasons** |
| --- | --- | --- |
| Pyrazinamide **(syrup, tablet, dispersible)** |  |  |
| Levofloxacin **(tablet, dispersible)** |  |  |
| Ethambutol **(tablet, dispersible)** |  |  |
| Linezolid **(syrup)** |  |  |
| Clofazimine **(gel capsule)** |  |  |
| Ethionamide **(tablet, syrup)** |  |  |
| Isoniazid **(tablet, syrup)** |  |  |
| Terizidone **(capsule, syrup)** |  |  |
| Delaminid **(tablet)** |  |  |
| Paser **(granules)** |  |  |
| Bedaquiline **(tablet)** |  |  |

1. **What would you tell other children who have to take medication for DR-TB?**
   1. Would you give them any ideas about how to make the treatment easier?
   2. What would you tell them not to do?
2. **Was it easier for you to take medicine in the hospital or at home?**

HOME: ______________ HOSPITAL: ______________

1. **Is there anything else that you would like to add or share with us today?**

_______________________________________________________________________________________________

Please remind your caregivers to sign the register for you ☺

Thank you so much for being a part of our group today. I had so much of fun hearing everyone’s stories and hearing of how you have become a **hero**.

You are a **Health Ninja Superhero**, because you took all your medication and beat the TB Germ! You are also a hero for learning everything that you did today!

***TEAM TO PASS OUT BOOKS TO THE CHILDREN***

Here are some books for you to take home, learn about TB and share this information with your friends and family back home ☺

***If there is still time, the facilitators can read out “TO BE… NOT TB” to the children in isiZulu.***

Annex 2:

**INTERVIEW/FOCUS GROUP DISCUSSION GUIDE FOR CAREGIVERS**

**FACILITATOR NAME & SURNAME: _________________________________**

**CO-FACILITATOR NAME & SURNAME: _____________________________**

**SCRIBE NAME & SURNAME: _____________________________________**

**Number of caregivers in the group: __________**

**Age group of children: ___________**

Good day everyone, welcome to our focus group meeting to improve the management of children with tuberculosis. My name is ***(Facilitator Name)***. I will be facilitating this session. Let me introduce you to ***(name of co-facilitator)*** who will be assisting me. This is ***(name of scribe)*** who will be writing some of the info down.

Is everyone comfortable with us speaking in isiZulu?

YES: ________ NO: ________

*If all yes, then speak in isiZulu. If some no, then mix with English and isiZulu.*

Please note that we will be taping the discussions. Once completed we will write down the common messages / experiences in the recording and analyze it to assist other children taking the medicine.

I hope everyone is ok. Thank you for joining us. Remember the Coronavirus is still around, so please make sure that you always use your mask, covering your nose and mouth and remain at least 1.5m apart. If your mask is loose or breaks during the course of the day, then please request for a new one from us.

**Before we get started, please can we make sure that everyone has signed the register when you came in and checked that all your details are correct on the register? If you have not done so yet, then please raise your hand and we will bring the register to you.**

Is there anyone who has been in a group where you were asked your opinions before? Please raise your hands.

*Co-facilitator to count number of hands out loud (1,2,3, 4….).*

*Facilitator to repeat final count and say these figures out loud for the Dictaphone:*

*FOR EXAMPLE “So…. there is 4 that did participate previously” and 6 that did not participate.*

*Scribe or Co-Facilitator to indicate how many YES: ______ and how many NO: _________ on the tool.*

**We are going to start with some basic instructions to enjoy our discussions**

1. Please put all phones on silent or off. If you need to take an URGENT call then please request to be excused from the group first.
2. Always remember that this is a safe space, and you all can speak openly and freely.
3. There are no wrong or right answers. We would just like to know what your thoughts and experiences were like.
4. Your answers will not be given or shown to anyone else, other than our study team.
5. As we are about to start this discussion, please can we allow everyone to have some input into the discussions.

**Are there any questions?**

Let’s get started ☺

1. Firstly, we will start by introducing ourselves. You don’t have to provide your real/ID name, but just one that we can call you by. You can also tell us one, quick thing that you would like everyone to know about you. I will start to give you an example

*HELLO EVERYONE, MY NAME IS _________________________ AND I WOULD LIKE YOU TO KNOW THAT I AM A* ***HEALTH NINJA*** *THAT FIGHTS FOR A BETTER TOMORROW FOR YOU AND FOR ME.*

**(5 minute introduction, including introduction of caregivers)**

1. Great, welcome again everyone. It is so nice to meet you all. Tell us a little bit about yourselves?

***IF THE GROUP DOES NOT TALK AT FIRST, THEN PROMPT THEM WITH THE POINTS IN RED***

- 1. *Occupation, pensioner*

*EMPLOYED________ UNEMPLOYED_________*

- 1. *Where do you live (rural, urban, how many people at home, etc)*

*RURAL _________ URBAN__________*

- 1. *No. of people at home­­­­­­­____________________*
  2. *What was the age of child when they started TB medication*?

AGE GROUPS: 0-5 ______________ 6-10 _____________ 7-14 ____________

1. **Tell me the story of how your child was diagnosed with DR-TB**
   1. What happened?
   2. When did you find out?
   3. How was the child told that he had DR-TB?
   4. Whom else did you tell that child has TB? Did you tell the family or school and how did they respond?
   5. How was the treatment by the healthcare workers?
   6. How were you informed about the hospitalization of your child to the big hospital?
2. **What have been the most difficult parts of the treatment journey?**
   1. What are some of the challenges to give your child the medicine at home/school or with your family?
   2. Did you miss or gain any opportunities (e.g. employment) due to caring for your child with TB?
3. **We now want to know your experiences with giving your child the medicine and how was it for your child to take the TB medicine? I am going to show you each of the medicine and the different ways it is available and you will tell us about your experience.**

**Refer to poster or flip chart with pictures of each medicine (box, blister back, packet in which the medicine is given to patient, label, syrup)**

Facilitator: Please say the name of the medication **AND** reference number out loud for the recorder (if they point to it)

- 1. **Show the adult formulations:** Adult medication are tablets that have to be broken, crushed and dissolved before giving the medicine to you child. Did you have to give adult medicine to your child? YES ________________ No _______________ (count and tell us how many said yea or no)

If yes, tell us about your experience (good or bad)

- 1. **Show the syrups:** Did you give your child syrups to treat the TB? If yes, tell us about your experience (good or bad).
  2. **Show the tablets:**

Did you give your child these tablets, syrups or dispersible (tablets that could be dissolved in a little bit of water)? If yes, tell us about your experience (good or bad).

| **Medicine** | **Good** | **Bad** |
| --- | --- | --- |
| Pyrazinamide **(syrup, tablet, dispersible)** |  |  |
| Levofloxacin **(tablet, dispersible)** |  |  |
| Ethambutol **(tablet, dispersible)** |  |  |
| Linezolid **(syrup)** |  |  |
| Clofazimine **(gel capsule)** |  |  |
| Ethionamide **(tablet, syrup)** |  |  |
| Isoniazid **(tablet, syrup)** |  |  |
| Terizidone **(capsule, syrup)** |  |  |
| Delaminid **(tablet)** |  |  |
| Paser **(granules)** |  |  |
| Bedaquiline **(tablet)** |  |  |

1. **What are some of the things that you did to make your child ready to take their medicines?**

Did you have to distract them or talk to the child and convince them to take the meds?

1. **Of all these medications which one did the child have difficulty with?**

**How did the child react to taking the medicine?**

**Did they spit /vomit out the medicine?**

Which of the medicine did the child have difficulty taking, did they spit / vomit it out?

*Show each of the pictures and count the hands.*

| **Medicine** | **YES** | **NO** |
| --- | --- | --- |
| Pyrazinamide **(syrup, tablet, dispersible)** |  |  |
| Levofloxacin **(tablet, dispersible)** |  |  |
| Ethambutol **(tablet, dispersible)** |  |  |
| Linezolid **(syrup)** |  |  |
| Clofazimine **(gel capsule)** |  |  |
| Ethionamide **(tablet, syrup)** |  |  |
| Isoniazid **(tablet, syrup)** |  |  |
| Terizidone **(capsule, syrup)** |  |  |
| Delaminid **(tablet)** |  |  |
| Paser **(granules)** |  |  |
| Bedaquiline **(tablet)** |  |  |

- 1. Did you have to bribe or force the child to take the medicine? Please explain
  2. Of all the medicine that the child took, which one’s did the child prefer?

(adult formulations, dispersible tablets or syrups)

ADULT_________ SYRUPS___________ DISPERSIBLE______

- 1. When or what time in the day did you normally give the child your medication? (any reminders e.g. phone, TV programme, etc)

1. **Is there anything you did to make it easier for the child to take their medications?**
   1. Why do you think this is the case?
   2. What do you normally do for the child to take the medication? (If no answers from the group, ask if the child was bribed, forced, restrained, etc to give the medication)
2. **How easy or difficult was it for you to prepare the medicine? Please Explain**

**USE THE MEDICINE BOARDS PROVIDED**

| **Medicine** | **Dispersible tablets** | **Tablets** | **Syrups** | **Gel Capsule** | **Granules** |
| --- | --- | --- | --- | --- | --- |
| Pyrazinamide |  |  |  | X | X |
| Levofloxacin |  |  | X | X | X |
| Ethambutol |  |  | X | X | X |
| Linezolid | X | X |  | X | X |
| Clofazimine | X | X | X |  | X |
| Ethionamide | X |  |  | X | X |
| Isoniazid | X |  |  | X | X |
| Terizidone | X | X |  |  | X |
| Delaminid | X |  | X | X | X |
| Paser | X | X | X | X |  |
| Bedaquiline | X |  | X | X | X |

1. **Where do you store the medications your child is taking?**
   1. Where? (place) and why?
   2. How do you store the medication?
   3. What is the reason?
2. **What would you tell other caregivers whose children are diagnosed with DR-TB about the experience?** Would you give them any special advice?
3. Thank you to all of your for coming and sharing your experiences with us. We hope that this was a good learning experience for all of us.

**Do you have any questions?**

**Thank you for sharing your experience with us. We appreciate it and hope that the information gathered here today can help us make child friendly formulations more accessible and treatment better outcomes.**

**Please remember that if you require a reimbursement of travel costs, then please remember to sign and collect your envelope.**

Annex 3

Tell me about the kind of work you do for children with drug-resistant TB:

Are you involved in preparing or administering the medications to children? If so, tell me more about what you do.

What do you think are the biggest challenges for children with DR-TB?

4. Describe specific challenges you have with giving the medications to the children. Do these challenges vary by age?

5. What do you think is the hardest part about giving the medications? Does this vary by age?

6. What do you do to make it easier for the children to take their medications? Does this vary by age?

7. What do you think are the biggest adherence challenges for children with DR-TB? Does this vary by age?

8. How do you think these challenges can be addressed?

9. If you could design the ideal medication for treating children with DR-TB, what would it be? Would this vary by age?

10. Is there anything else you would like to tell me about the medications used for treating children with DR-TB?
